# Prevalence and Distribution of Dengue Virus in *Aedes aegypti* in Yogyakarta City before Deployment of Wolbachia Infected *Aedes aegypti*

**DOI:** 10.3390/ijerph16101742

**Published:** 2019-05-16

**Authors:** Ayu Rahayu, Utari Saraswati, Endah Supriyati, Dian Aruni Kumalawati, Rio Hermantara, Anwar Rovik, Edwin Widyanto Daniwijaya, Iva Fitriana, Sigit Setyawan, Riris Andono Ahmad, Dwi Satria Wardana, Citra Indriani, Adi Utarini, Warsito Tantowijoyo, Eggi Arguni

**Affiliations:** 1 Centre of Tropical Medicine, World Mosquito Program Yogyakarta, Faculty of Medicine, Public Health and Nursing, Universitas Gadjah Mada, Yogyakarta 55281, Indonesia; ayu.rahayu@worldmosquito.org (A.R.); utari.saraswati@worldmosquito.org (U.S.); endah.supriyati@worldmosquito.org (E.S.); aruni.dian@gmail.com (D.A.K.); rio.hermantara@yahoo.com (R.H.); anwar.rovik@worldmosquito.org (A.R.); m.edwin.d@ugm.ac.id (E.W.D.); iva.fitriana@worldmosquito.org (I.F.); sigit.setyawan@worldmosquito.org (S.S.); riris.andono@worldmosquito.org (R.A.A.); satria.wardana@worldmosquito.org (D.S.W.); citra.indriani@worldmosquito.org (C.I.); adi.utarini@worldmosquito.org (A.U.); warsito.tantowijoyo@worldmosquito.org (W.T.); 2 Department of Epidemiology, Biostatistics and Population Health, Faculty of Medicine, Public Health and Nursing, Universitas Gadjah Mada, Yogyakarta 55281, Indonesia; 3 Department of Health Policy and Management, Faculty of Medicine, Public Health and Nursing, Universitas Gadjah Mada, Yogyakarta 55281, Indonesia; 4 Department of Child Health, Faculty of Medicine, Public Health and Nursing, Universitas Gadjah Mada, Yogyakarta 55281, Indonesia

**Keywords:** dengue, *Aedes aegypti*, prevalence, Yogyakarta City, world mosquito program

## Abstract

Indonesia is one of the countries where dengue infection is prevalent. In this study we measure the prevalence and distribution of dengue virus (DENV) DENV-infected *Aedes aegypti* in Yogyakarta City, Indonesia, during the wet season when high dengue transmission period occurred, as baseline data before implementation of a Wolbachia-infected *Aedes aegypti* trial for dengue control. We applied One-Step Multiplex Real Time PCR (RT-PCR) for the type-specific-detection of dengue viruses in field-caught adult *Aedes aegypti* mosquitoes. In a prospective field study conducted from December 2015 to May 2016, adult female *Aedes aegypti* were caught from selected areas in Yogyakarta City, and then screened by using RT-PCR. During the survey period, 36 (0.12%) mosquitoes from amongst 29,252 female mosquitoes were positive for a DENV type. In total, 22.20% of dengue-positive mosquitoes were DENV-1, 25% were DENV-2, 17% were DENV-3, but none were positive for DENV-4. This study has provided dengue virus infection prevalence in field-caught *Aedes aegypti* and its circulating serotype in Yogyakarta City before deployment of Wolbachia-infected *Aedes aegypti*.

## 1. Introduction

Dengue is the fastest spreading mosquito borne disease in the world and is endemic in most tropical countries with an estimated 96 million symptomatic cases annually [1]. According to World Health Organization (WHO) (2009) an estimated 2.5 billion people live in areas where there is a risk of dengue transmission, with about 975 million of those living in built-up areas in different countries located tropically and sub-tropically in Southeast Asia, the Americas and the Pacific globally [2]. The incidence of dengue virus infection is much greater in Asian countries than in other regions. The latestestimation showed an increasing number of dengue deaths, from 8000 in 1992 to 11,000 in 2010, making dengue one of the important arbovirus infection globally [3].

The causative agents of dengue viruses are members of the *Flaviviridae* family, designated DENV-1, DENV-2, DENV-3, and DENV-4 [4]. DENV are transmitted primarily by *Aedes aegypti* with *Aedesalbopictus* regarded as a sondary vector. Several factors have been implicated in the global resurgence of dengue, including the failure to control the *Aedes* populations, uncontrolled urbanization, and an unprecedented population growth [5]. The seasonal dynamics of *Aedes aegypti* population size commonly have positive association with climate variables such as temperature and more rainfall [6], and also relative humidity [7]. The vector presents in two distinct stages: aquatic with development phases of egg, larva and pupa, and terrestrial, which corresponds to adult mosquitoes. Both stages are subject to environmental and climate changes [8].

Indonesia is one of the largest countries in the Asian Pacific region with a population of 245 million, with almost 60% of the people living on the island of Java, which is most severely afflicted by periodic outbreaks of dengue [9]. Epidemic dengue hemorrhagic fever (DHF) is well documented in Indonesia and was first recognized in 1968 on the island of Java [10]. Dengue epidemiology in Indonesia has been described mostly in the form of case series, reporting on single outbreaks, or clinical and virological studies on DHF [11]. Periodic outbreaks have occurred in Indonesia with an increasing number of cases and severity [12]. All four serotypes of DENV have been found to be circulating in several cities in Indonesia, including Yogyakarta, with DENV3 predominating [9,13,14,15]. The Surveillance and Data Centre of the Indonesian Ministry of Health (2016) stated that in 2015, Yogyakarta Province was one of the most prevalent provinces in Indonesia for dengue hemorrhagic fever cases with incidence rate 92.96/100,000 [16]. According to Yogyakarta Province Health Office, Yogyakarta City, is one of dengue endemic district area in Yogyakarta Province, with an incidence rate (IR) of 352 per 100,000 persons in 2016, and 81 per 100,000 persons in 2017. The emerging pattern of this disease provoked our interest to study the prevalence of the dengue virus in mosquitoes in Yogyakarta city and to map the prevalence of dengue infection presently found in this region of Indonesia. As the first randomized trial of Wolbachia as dengue control commenced in Yogyakarta City in 2017 [17], our data of circulating dengue virus in *Aedes aegypti* will give important baseline data for future vector control intervention study.

## 2. Materials and Methods

### 2.1. Study Area

**Figure 1:**
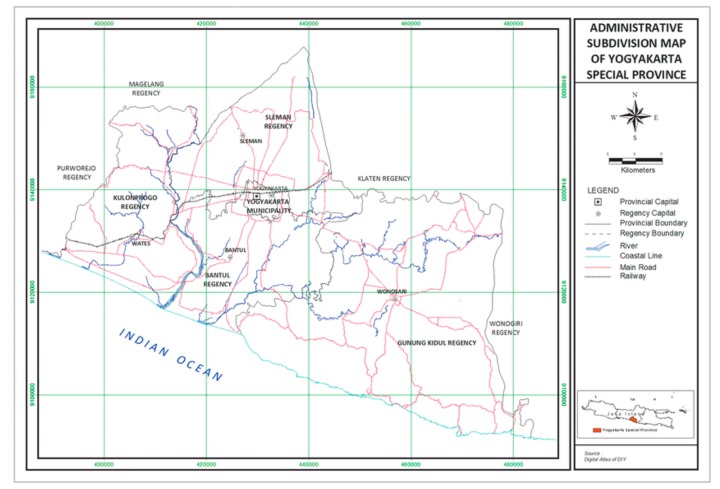
Map of Yogyakarta Special Province.

Yogyakarta City (Figure 1) is the capital of Yogyakarta Province, located in south central part of Java. Yogyakarta has a tropical climate. Data from Yogyakarta Office of Statistic (2016) recorded that the wet period started from December. Yogyakarta, which lies between 7.33′–8.12′ South latitude and 110.00′–110.50′ East longitude of Greenwich, has an area 3185.80 km^2^ or 0.17 percent of Indonesia’s land area (1,860,359.67 km^2^). The average temperature in Yogyakarta during the period of December 2015 and May 2016 was recorded at 27 °C which was higher than the average temperature during 2014 (which was recorded at 26.3 °C) with the minimum temperature of 20 °C and the maximum temperature of 33.3 °C. Precipitation was recorded at 280 mm and rain days per month was an average of 11 times. Humidity was recorded at an average of 88% [18] (Table 1).

### 2.2. Entomological Surveillance

**Figure 2:**
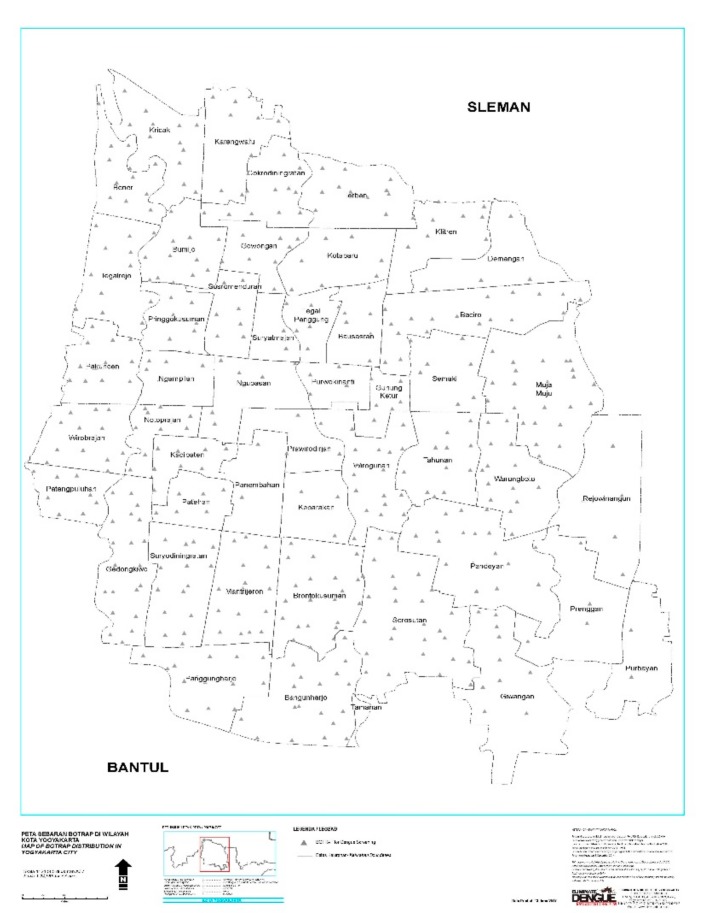
Map of the study areas. BG-trap location in Yogyakarta City.

Wild adult mosquitoes were collected using BioGent Sentinel (BG) traps (Biogents AG, Regensburg, Germany) from December 2015 to May 2016. These traps were put inside houses and operating 24 h/7 days during study period with connection of main electricity power supply and also have battery as back up system. One BG-trap was each set in a grid of 250 × 250 m^2^. The total numbers of BG-traps was 437, which were distributed across each neighborhood (Figure 2). Samples were collected weekly. Adult *Aedes aegypti* were identified and separated by sex morphologically and the presence of DENV were tested in female mosquitoes using RT PCR.

### 2.3. RNA Extraction and RT-PCR Assays

Viral RNA was extracted from adult female mosquitoes by using 96-well plates. The mosquitoes were suspended in 100 ul of mix buffer (squash buffer and Proteinase-K) then grinded by using bead beater machine. The pellet was sedimented by centrifugation at 3000 rpm for 2 min. The RNA was extracted by boiling method using Biorad C1000 with protocols; 58 °C for 5 min, 96 °C for 5 min, and 12 °C for 5 min. Samples were tested into two round PCR. The first round was aimed to determine positive DENV using dengue general primer (Table 2). Every 24 mosquito’s RNA were collected into one PCR sample. The first One-Step Multiplex Real Time PCR was performed by a RT step at 50 °C for 10 min and a denaturation step at 95 °C for 30 s, followed by 45 cycles of 95 °C for 3 s, 60 °C for 30 s, 72 °C for 1 s, and a final period of 37 °C for 30 s. In the cases where DENV was detected in a pool of mosquitoes, trace-back PCR was performed to determine the individual mosquitoes with DENV infection. Once the individual positive DENV mosquito was confirmed, this sample was tested in the sond round using One-step multiplex Real Time-PCR with the primers and probes (Table 2) to detect DENV serotypes 1–4 [19]. The PCR reaction was performed by RT step at 61 °C for 10 min and a denaturation step at 95 °C for 2 min, followed by 45 cycles of 95 °C for 15 s, 60 °C for 30 s, and a final period of 37 °C for 1 min. Positive control (standard curve) and negative control (water) were included in each RT PCR. The positive result was confirmed by the amplification sigmoid curve in RT PCR analyses using a Light Cycler machine (Roche, Germany).

### 2.4. Dengue Surveillance

Data on notified dengue cases were obtained from District Health Office Yogyakarta City passive surveillance system within the same period. The system is hospital-based and relies on clinical definition of dengue infection, which include only those classified as DHF or DSS as per 2011 revised national guideline.

### 2.5. Ethics Statement

Written informed consent was obtained from an adult head of household for entomological surveys, and was documented upon obtaining access to the household. Information sheet of data collection procedures were provided to each household. The Ethical Review Board of the Faculty of Medicine, Public Health and Nursing, Universitas Gadjah Mada approved this study, with the approval number KE/FK/818/EC.

### 2.6. Data Sharing

Information about dengue cases described in this manuscript is part of the data bank of the Yogyakarta City Health Office. Access to the data can be obtained by contacting Surveillance Division of Yogyakarta City Health Office.

## 3. Results

### 3.1. Adult Aedes aegypti Population Density and Prevalence of DENV-Infected Mosquitoes

During five months of study period, a total of 29,252 female *Aedes aegypti* were captured in this study (Table 3). The maximum number of *Aedes aegypti* mosquito population in BG-traps during the period was registered in January 2016. From all 437 BG-traps, 19 were containing dengue-positive mosquitoes during February to May 2016. The average number of mosquitoes was four mosquitoes per trap per week. Average positivity rate was up to one per trap per week (data not shown). In total, 36 DENV-infected *Aedes aegypti* were detected with a range of Ct values from 19–30. The number of female *Aedes aegypti* captured was not significantly correlated with the number of DENV-infected *Aedes aegypti* (Table 3). The highest percentage of DENV-infected *Aedes aegypti* was recorded in March 2016.

### 3.2. Monthwise DENV Prevalence in Trapped-Mosquito and Dengue Cases Reported in Human

**Figure 3:**
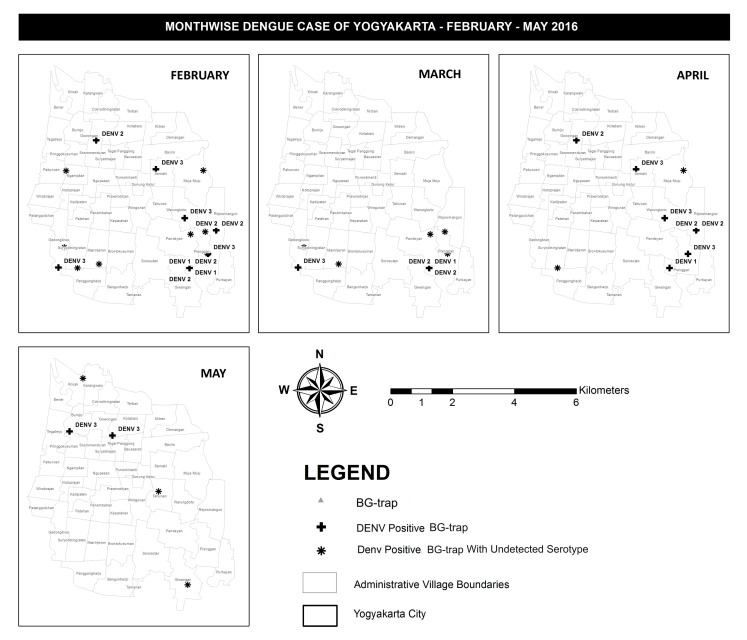
Monthly data depicting DENV infected mosquitoes trapped in the study area.

Figure 3 describes the spatial distribution of DENV infected *Aedes aegypti* in 19 BG-traps in Yogyakarta City during the study period. From December 2015 to January 2016 no DENV positive *Aedes aegypti* were detected. In February 2016, a total of one DENV positive *Aedes aegypti* was detected in Prenggan village. In March 2016, a total of 11 DENV positive *Aedes aegypti* were detected in the villages of Bumijo, Kotabaru, Ngampilan, Muja-muju, Tahunan, Gedongkiwo, Mantrijeron, and Prenggan. In April 2016, a total of three DENV positive *Aedes aegypti* in the villages of Warungboto, Giwangan, and Prenggan. In May 2016, a total of four DENV positive mosquitos in village of Kricak, Sosromenduran, Baciro, and Rejowinangun. Interestingly, in the same area of Prenggan village DENV-positive *Aedes aegypti* were trapped 3 months consecutively, February, March, and April (Figure 3).

### 3.3. Dengue Virus Prevalence in Aedes aegypti-Captured Mosquito and Its Relation to Notified Dengue Cases

**Figure 4:**
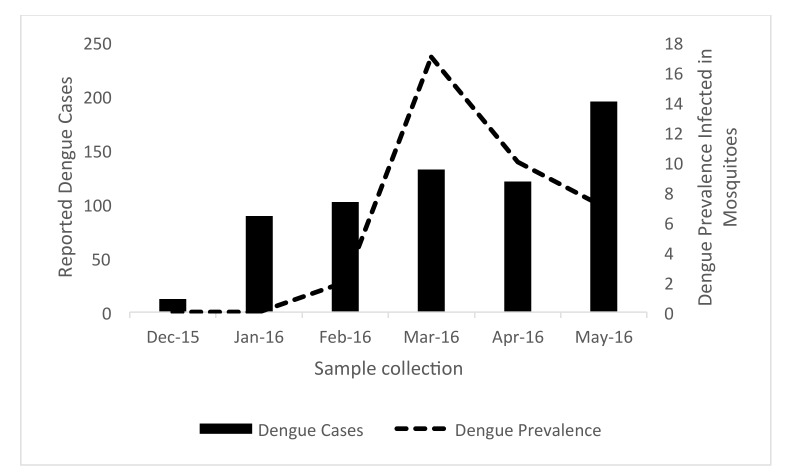
Notified dengue cases (bars) and prevalence of DENV infected mosquitoes through the study period (December 15–May 16).

We explored whether there was a temporal correlation between the prevalence of DENV infected *Aedes aegypti* and notified dengue cases reported by district health office (Figure 4). Twelve cases (IR 0.27/10,000) were reported from Yogyakarta City District Health Office during December 2015, and an increasing number were reported later. In January 2016, 89 cases were reported (IR 2.15/10,000), 102 cases (IR 2.46/10,000), 132 cases (IR 3.19/10,000), 121 cases (IR 2.92/10,000) and 195 cases (IR 4.71/10,000), consecutively until May 2016.

### 3.4. DENV Serotypes in Aedes aegypti

**Figure 5:**
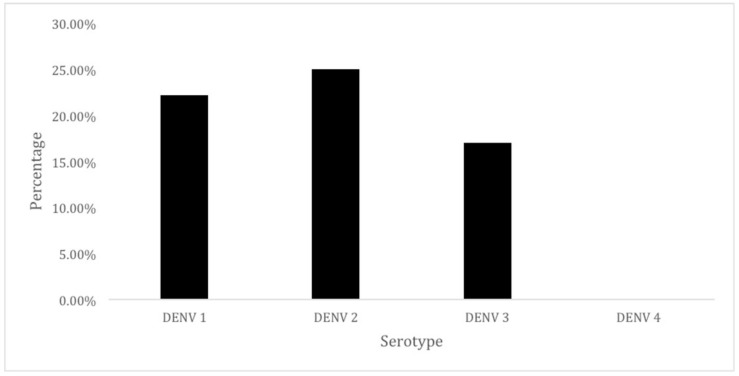
The distribution of DENV serotypes detected in infected female mosquitoes.

To determine the circulating DENV serotype in captured *Aedes aegypti* in city of Yogyakarta, serotyping was performed using one-step multiplex real time RT-PCR assay. Using this method, 23 of 36 samples positive dengue were successfully serotyped. The predominant serotype circulating in city of Yogyakarta was DENV-2 (25%), followed by DENV-1 (22.20%), and DENV-3 (17%). DENV-4 serotype was not detected (Figure 5).

## 4. Discussion

In the absence of commercially available antiviral, and even though progress on developing a cost-effective for mass immunization of dengue vaccine is promising, the prevention and control of dengue infection relies upon control of the vector mosquitoes *Aedes aegypti* [20,21,22,23]. Many dengue endemic countries use entomological surveys as the routine method recommended by WHO to record mosquito populations [2]. Historically, rigorously applied vector control couldsucceed in reducing dengue transmission in several countries, but they were not sustainable, thus the problem persisted [24,25]. In Indonesia, and mostly other developing countries, the vector control strategy has focused mainly on elimination of *Aedes* larval breeding habitats by water container cleaning (closing, washing, dumping) and insticide space spraying against adult mosquitoes during outbreaks [21,26].

Molecular detection of dengue virus has been used for epidemiological surveillance as a sensitive tool for assessing vector infection [27,28,29]. We used rapid diagnostic RT-PCR for detection and typing dengue viruses in adult female *Aedes aegypti* mosquitoes field population. In this system, prevalence of dengue virus in female *Aedes aegypti* mosquito was 0.12%, averagely distributed in the whole study area, reflecting the wide distribution of dengue viruses in Yogyakarta City. This prevalence was lower compared to 6.91% in Singapore [30] and 17.7% in endemic area in India [31]. Basic reproduction numbers, spatial heterogeneity of *Aedes aegypti* breeding, and the effect of climate are considered as important factors in dengue virus infection in mosquitos [32], as well as the specific genotype of mosquito [33,34,35]. The extrinsic incubation period (EIP) of the dengue virus together with high plasma viremia level of humans and 50% mosquito infectious dose (MID_50_) are important factors for dengue infection in mosquitoes [36,37,38].

In this study, we showed that infection status of dengue virus in *Aedes aegypti* population is not correlated with mosquito density. This idea also has never been demonstrated by other reported studies [39,40]. Even though an increase in the adult mosquito population would be thought have a positive correlation with threat of dengue infection to the community, the number of infected female mosquitoes is more important, rather than the total adult population size. It is also interesting to discover relationships between number of infected female mosquito and the dengue cases in human in a longer prospective study. However, since only severe forms requiring hospitalization were recorded in district health office, asymptomatic and sub clinical patients remain uncaptured by our current passive surveillance system. Thus, the true dengue burden in Yogyakarta City has tobe critically analyzed.

Between December 2015 and May 2016, the predominant serotype in field-caught *Aedes aegypti* in Yogyakarta City was DENV-2. Data of predominance DENV-3 *Aedes aegypti* population has been reported in Bantul District, Yogyakarta [41]. The predominance of DENV-2 has been described previously in Yogyakarta in 1995 from serum sample from patients [13]. During our study period, we did not detect any DENV-4 in caught-female *Aedes aegypti*; however, circulating DENV-4 was reported in recent study of dengue infection in human population of Yogyakarta City in 2012–2015 [42]. The absence of routine data surveillance of dengue virus serotype in serum patient makes this study difficult to look correlation between the co-circulating dengue serotype and clinical severity.

In our study, there are samples that could be detected using general dengue primer, but failed to be serotyped. The variation of dengue viral RNA from each field-caught mosquito (i.e., method of RNA extraction, preservation of samples, virus titer) and the specificity of serotyping primers are the factors that need to be considered. A high performance detection system is very important to have a proper estimation of dengue virus infection rates. We used primers reported by Hue and co-workers [19] for detecting and classifying dengue virus in mosquitoes. In this respect, this detection system needs to be studied more.

The molecular technique of RT-PCR shows dengue virus serotypes, which are currently circulating within the certain area. The change in serotype may indicate a forth coming outbreak of more severe dengue cases, as weaning of antibodies against the new circulating dengue serotype may occur. Since viremic patients can transmit dengue to *Aedes aegypti* [36], the circulating serotype in humans may reflect the similar pattern of dengue serotype in the mosquito, and vice versa.

The monitoring of the dengue virus type(s) infecting *Aedes aegypti* mosquitoes may complement the current virology surveillance for dengue outbreaks [43]. Understanding of dengue outbreaks in Indonesia and developing risk management scenarios are important keys to vector control and prevention of dengue transmission. One of the novel bio-control strategies to reduce the capability of dengue virus transmission is to introduce Wolbachia, the endosymbiotic bacterium, into *Aedes aegypti* population [44,45]. Field trials in Australia and Yogyakarta, Indonesia, indicated the establishment of wMel strain of Wolbachia for years [45]; submission for publication]. Study from Australia indicated that after one year of establishment in wild population of *Aedes aegypti*, Wolbachia-mediated dengue interference is not changed [46]. Even though the mechanism of dengue blocking mechanism is not well understood yet, it is predicted that evolution of DENV will be likely to occur in order to reduce complete blocking by Wolbachia [47]. The different serotypes may also have different defense pathways to Wolbachia’s blocking mechanism, thus the circulating DENV serotype may display alternative responses to by Wolbachia introduction in natural populations of *Aedes aegypti*.

Structured implementation trials to assess the epidemiological impact of this method have been started. To the best of our knowledge, this current study is the first study reporting dengue virus infection rate in *Aedes aegypti* population in the wet season, captured over a wide area in Yogyakarta City. Our results showed the preliminary data of DENV circulating in the *Aedes aegypti* mosquito population in Yogyakarta City. The first randomized trial of Wolbachia-infected *Aedes aegypti* in Yogyakarta City in 2017 [17] may impact local dengue transmission and dengue virus serotype pattern, thus monitoring of dengue virus infection in mosquitoes should be periodically done.

## 5. Conclusions

This study may act as important baseline data of circulating dengue virus serotypes in Yogyakarta City before the implementation of Wolbachia technology. As a promising candidate for dengue transmission control, Wolbachia-infected *Aedes aegypti* may have an impact on the pattern of circulating dengue virus serotypes. Future study needs to address the role of environmental dynamics and dengue virus in areas dominated with Wolbachia-infected *Aedes aegypti*.

## Figures and Tables

**Table 1:** Climatic conditions in Yogyakarta.

Month	Temperature (°C)	Humidity (%)	Precipitation (mm)
Dec-15	26.9	88	459
Jan-16	27.5	86	152
Feb-16	26.5	89	323
Mar-16	26.8	89	425
Apr-16	27.3	88	184
May-16	27.2	88	137

**Table 2:** Primer and probe sequences.

Primer/Probe	Primer/Probe Sequences
DENV-Forward	5′-AAGGACTAGAGGTTAGAGGAGACCC-3′
DENV-Reverse	5′-CGTTCTGTGCCTGGAATGATG-3′
DENV-Probe	5′FAM- AACAGCATATTGACGC TGGGAGAGACCAGA-3′BHQ1
DENV-1-Forward	5′-ATCCATGCCCAYCACCAAT-3′
DENV-1-Reverse	5′-ATGTGGGTTTTGTCCTCCAT-3′
DENV-1-Probe	5′FAM-TCAGTGTGGAATA GGGTTTGGATAGAGGAA-3′BHQ1
DENV-2-Forward	5′-TCCATACACGCCAAACATGAA-3′
DENV-2-Reverse	5′-GGGATTTCCTCCCATGATTCC-3′
DENV-2-Probe	5′FAM-AGGGTGTGGATTCGAGAA AACCCATGG-3′BHQ1
DENV-3-Forward	5′-TTTCTGCTCCCACCACTTTC-3′
DENV-3-Reverse	5′-CCATCCYGCTCCTTGAGA-3′
DENV-3-Probe	5′Cyan500-AAGAAAGTTGGTAGTT CCCTGCAGACCCCA-3′BHQ1
DENV-4-Forward	5′-GYGTGGTGAAGCCYCTRGAT-3′
DENV-4-Reverse	5′-AGTGARCGGCCATCCTTCAT-3′
DENV-4-Probe	5′Cyan500-ACTTCCCTCCTCTTYTT GAACGACATGGGA-3′BHQ1

**Table 3:** Number of captured *A. aegypti* and number that tested positive for DENV infection.

Month	Number of Total *Aedes aegypti*	Number of Female *Aedes aegypti* (%)	Number of Females with DENV Infection (%)
Dec-15	6128	4258 (69.48)	0 (0.00)
Jan-16	11,034	8528 (77.29)	0 (0.00)
Feb-16	6524	5065 (77.64)	2 (0.04)
Mar-16	5419	4236 (78.17)	17 (0.40)
Apr-16	5967	4986 (83.56)	10 (0.20)
May-16	2649	2179 (82.26)	7 (0.32)
Total	37,721	29,252 (77.55)	36 (0.123)
